# Discriminative ability of the generic and condition-specific Child-Oral Impacts on Daily Performances (Child-OIDP) by the Limpopo-Arusha School Health (LASH) Project: A cross-sectional study

**DOI:** 10.1186/1471-2431-11-45

**Published:** 2011-05-26

**Authors:** Hawa S Mbawalla, Matilda Mtaya, Joyce R Masalu, Pongsri Brudvik, Anne N Astrom

**Affiliations:** 1 Department of Clinical Dentistry, Community Dentistry, University of Bergen, Bergen, Norway; 2 Centre for International Health, University of Bergen, Bergen, Norway; 3 Muhimbili University of Health and Allied Sciences, Dar Es Salaam, Tanzania; 4 Department of Clinical Dentistry-Orthodontics, University of Bergen, Bergen, Norway

## Abstract

**Background:**

Generic and condition-specific (CS) oral-health-related quality-of-life (OHRQoL) instruments assess the impacts of general oral conditions and specific oral diseases. Focusing schoolchildren from Arusha and Dar es Salaam, in Tanzania, this study compared the discriminative ability of the generic Child OIDP with respect to dental caries and periodontal problems across the study sites. Secondly, the discriminative ability of the generic-and the CS Child OIDP attributed to dental caries, periodontal problems and malocclusion was compared with respect to various oral conditions as part of a construct validation.

**Methods:**

In Arusha, 1077 school children (mean age 14.9 years, range 12-17 years) and 1601 school children in Dar es Salaam (mean age 13.0 years, range 12-14 years) underwent oral clinical examinations and completed the Kiswahili version of the generic and CS Child-OIDP inventories. The discriminative ability was assessed as differences in overall mean and prevalence scores between groups, corresponding effect sizes and odd ratios, OR.

**Results:**

The differences in the prevalence scores and the overall mean generic Child-OIDP scores were significant between the groups with (DMFT > 0) and without (DMFT = 0) caries experience and with (simplified oral hygiene index [OHI-S] > 1) and without periodontal problems (OHI-S ≤ 1) in Arusha and Dar es Salaam. In Dar es Salaam, differences in the generic and CS Child-OIDP scores were observed between the groups with and without dental caries, differences in the generic Child-OIDP scores were observed between the groups with and without periodontal problems, and differences in the CS Child-OIDP scores were observed between malocclusion groups. The adjusted OR for the association between dental caries and the CS Child-OIDP score attributed to dental caries was 5.4. The adjusted OR for the association between malocclusion and CS Child-OIDP attributed to malocclusion varied from 8.8 to 2.5.

**Conclusion:**

The generic Child-OIDP discriminated equally well between children with and without dental caries and periodontal problems across socio-culturally different study sites. Compared with its generic form, the CS Child-OIDP discriminated most strongly between children with and without dental caries and malocclusion. The CS Child OIDP attributed to dental caries and malocclusion seems to be better suited to support clinical indicators when estimating oral health needs among school children in Tanzania.

## Background

Planning dental treatment within a public health system requires information on the prevalence and distribution of oral diseases [1]. However, normative treatment needs, reflected in clinical oral indicators, provide little information about the patients' self-perceived treatment needs. To overcome this limitation, oral-health-related quality-of-life (OHRQoL) instruments have been developed to assess the impact of oral health on daily life activities [2]. According to Locker [3], the subjective perception of oral health and treatment needs is considered to be the consequence of oral conditions, although studies that have investigated the relationship between subjective and clinical oral health indicators have shown both strong and weak significant associations and even the absence of any relationship [4]. Numerous studies have identified a gap between professionally and self-defined oral health, suggesting that they document different dimensions of the human experience, which are conceptually and often empirically distinct, with different implications for treatment need [5]. Consequently, OHRQoL instruments are recommended to supplement clinical measures and as adjuncts to them [4].

Whereas clinical oral health indicators refer to specific oral conditions, such as dental caries, periodontal disease, and malocclusion, most OHRQoL indicators are generic in that they assess the overall impact of oral problems by considering numerous oral conditions. In contrast, condition-specific (CS) OHRQoL measures focus on particular diseases, conditions, symptoms, functions, or populations, and should be used when any of these attributes must be assessed [1]. CS instruments provide information about the consequences of a specific, untreated oral condition and the corresponding benefits of its treatment. This might make CS instruments more sensitive to small but clinically relevant changes in oral diseases than both generic HRQoL and OHRQoL instruments [1,6]. Assuming that oral conditions have consequences for more widespread health issues, Allen et al. [7] compared the validity of the Oral Health Impact Profile (OHIP) with a generic HRQoL instrument, SF36, in edentulous patients seeking implants or conventional dentures. Whereas OHIP discriminated between three clinically disparate groups, SF36 did not. Lee et al. [8] compared the performances of the Pediatric Quality of Life Inventory and the Early Childhood Oral Health Impact Scale and showed that the latter instrument was superior in identifying those children affected by early childhood caries from those without caries. However, with few exceptions, the superiority of CS measures to generic HRQoL and OHRQoL instruments has yet to be established [1,9-11].

One of the most commonly used OHRQoL instruments, the Oral Impact on Daily Performances (OIDP), is designed to be used both as a generic and a CS instrument. As a CS instrument, it can link specific oral conditions to an individual's quality of life [11]. The Child-OIDP [12], derived from the adult OIDP version, has been shown to be applicable to school children across occidental and non-occidental socio-cultural contexts, when used as self-administered questionnaires or in face-to-face interviews [for a review, see [13]]. However, there is little empirical evidence about the relationship between the Child-OIDP and various oral diseases or on whether those relationships vary across socio-cultural contexts. Few studies have compared the capacities of the generic and CS Child-OIDP inventories to discriminate between groups with different levels of normative treatment needs, as part of a construct validity assessment [14].

In Tanzania, dental diseases have remained at moderate levels, and approximately 30%-40% of the population, irrespective of age, is reportedly free of dental caries. However, Tanzanian children have for many years demonstrated a high prevalence of untreated dentinal lesions, with a majority located in molars, which show relatively slow progression [15]. Recently, 19.2% of a sample of rural school children was identified with normative treatment needs for dental caries [16]. Periodontal problems have been reported to account for 80% of all oral diseases in the Tanzanian population [17]. Poor oral hygiene at an age of 15 years or older is very common (65%-99%) and the prevalence of gingivitis is reported to range from 80% to 90% [18,19]. Previous studies have indicated a wide variation in the prevalence of malocclusion, ranging from 45% to 97% among school children [20]. Exposure to dental services is low in this country, particularly in rural areas, and dental pain and discomfort have been cited as common reasons for seeking dental care [17]. Information is needed about the generic and CS impacts of periodontal disease, dental caries, and malocclusion on children's quality of life, to guide the assessment of the dental treatment needs of Tanzanian school children.

### Purpose

Focusing on school children, this study compared the discriminative ability of the generic Child-OIDP for dental caries and periodontal problems across socio-culturally different study sites (Arusha and Dar es Salaam) in Tanzania. The discriminative ability of the generic and CS Child-OIDP attributed to dental caries, periodontal problems, and malocclusion were then compared with respect to various oral conditions among school children in Dar es Salaam, as part of a construct validation.

## Methods

### Arusha site

As a part of the Limpopo-Arusha school health project (LASH), a cross sectional study was performed in 2009 in Arusha, northern Tanzania, focusing on secondary school students. In this study area, the fluoride concentration in the drinking water has been estimated to be 3.6 mg/L [21]. Fifty-nine public secondary schools were listed, 31 of which fulfilled the inclusion criteria of being a public school with a student enrolment of more than 200. A one-staged stratified cluster design was utilized, with the school as the primary sampling unit. All available students in forms I and II (i.e. the two first school years) in 20 selected schools (10 urban and 10 rural) were invited to participate in the study. Ultimately, 1163 and 1249 students from urban and rural schools, respectively, were included in the study (2412/2988 participation rate, 80.7%). A structured questionnaire, including 165 questions, was initially developed in English, translated into Kiswahili, and then back-translated into English by independent translators qualified in English and Kiswahili. This questionnaire was completed by the students in a classroom setting under the supervision of trained research assistants. In total, 1077 of the 1331 participants (participation rate, 80.9%) enrolled in a random sub-sample of 10 schools (five urban and five rural) consented to undergo a full-mouth clinical oral examination. A sample size of 1200 school children was calculated to be sufficient for two-sided tests, assuming the prevalence of oral impact to be 0.40 and 0.50 in children with and without an orthodontic anomaly, respectively, a significance level of 5%, power of 90%, and a design factor of 2 [22]. The sampling procedure has been described in detail elsewhere [23]. Parents and students gave their written informed consent to participate in both the main questionnaire survey and the clinical examination. Permission to conduct the study was granted by the school authorities and the Ministries of Education and Health of Tanzania. Ethical approval was given by Muhimbili University of Health and Allied Sciences, the National Institutes for Medical Research in Tanzania and the Regional Committee for Research Ethics of Western Norway (REK Vest).

### Dar es Salaam site

A cross-sectional survey was conducted in 2006 in Dar es Salaam, the commercial capital of Tanzania. Dar es Salaam is divided into three districts, and two of them, Kinondoni and Temeke, are quite diverse in their socio-demographic profiles: Kinondoni has higher employment and literacy rates, and a greater proportion of the population uses electricity (the most expensive energy source) for cooking [24]. All districts have drinking water with a fluoride content of about 1 mg/L (1 ppm). The study population comprised children attending grade 7 (i.e the last school year) in public primary schools. A stratified proportionate two-staged cluster sampling design was utilized, with public primary schools as the primary sampling unit. A sample size of 1200 school children aged 12-14 years was calculated to be sufficient for two-sided tests, assuming the prevalence of oral impact to be 0.40 and 0.50 in children with and without an orthodontic anomaly, respectively, a significance level of 5%, power of 90%, and a design factor of 2 [22]. In total, 1601 children completed the clinical oral examination and a structured interview in the school setting. The interview schedule was developed in English and translated into Kiswahili by two trained research assistants. Oral health professionals reviewed the interview schedules for semantic, experiential, and conceptual equivalence. Sensitivity to culture and the selection of appropriate words were considered. The interview schedule was piloted before its administration. Informed consent was obtained from parents and students. Ethical approval was obtained from all the relevant persons, authorities, and committees in Tanzania and from the Regional Committee for Research Ethics of Western Norway (REK Vest). For a more detailed description of the sampling procedure, see [20].

### Variables and measurements

Identical variables were assessed at both study sites in terms of *socio-demographic factors*: age, sex, place of residence, and religious affiliation. *Oral-health-related quality of life *was measured using a Kiswahili version [20] of the eight-item generic and CS Child-OIDP inventories (e.g., during the preceding three months, how often have you had problems with your teeth and mouth that caused you difficulty with: eating, speaking, cleaning your teeth, smiling, sleeping, emotional balance, study, or social contact). Each item was scored on a scale of 0-3, which equated to (0) never, (1) once or twice a month, (2) once or twice a week, and (3) every day/nearly every day. The generic Child-OIDP was assessed at both study sites, whereas the CS Child-OIDP was assessed only in Dar es Salaam. The generic and CS Child-OIDP simple count (SC) scores were calculated by summing the dichotomized frequency items of (1) affected (original score 1-3) and (0) not affected (original score 0). The participants in Dar es Salaam were also asked to identify from a list of oral problems those that they believed caused the specific impact. The prevalence of generic and CS oral impact was calculated as the percentage of children with overall generic and CS Child-OIDP SC scores above zero. The CS Child-OIDP assessed only those impacts related to the specific oral conditions linked to various types of treatment needs. CS impacts related to toothache were considered to be CS Child-OIDP attributed to dental caries, whereas CS impacts related to swollen gums, bleeding gums, and ulcerous gums were considered CS Child-OIDP attributed to periodontal problems. Finally, CS impacts related to spaces between the teeth and bad positioning of the teeth were considered CS Child-OIDP attributed to malocclusion.

### Clinical oral examination

Clinical oral examinations were carried out at each site by one trained and calibrated dentist, together with dental assistants. Caries experience was assessed under field conditions and scored according to the criteria described by the World Health Organization [25]. Oral hygiene was assessed using the simplified Oral Hygiene Index (OHI-S) [26]. Plaque was assessed on six index teeth in terms of (0) no debris present, (1) soft debris covering more than one-third of the tooth surface, (2) soft debris covering more than one-third but not more than two-thirds of the tooth surface, or (3) soft debris covering more than two-thirds of the tooth surface. Calculus was assessed on six index teeth and recorded as (0) no calculus present, (1) supra-gingival calculus covering at most one-third of the tooth surface, (2) supra-gingival calculus covering more than one-third but not more than two-thirds of the tooth surface, or (3) supra-gingival calculus covering more than two-thirds of the tooth surface. For each individual, the debris and calculus scores for each index tooth were summed and divided by the number of teeth assessed (range 0-3). The average debris score was dichotomized into 0/1 = good/bad debris score (cut-off point 0.7). The average calculus score was dichotomized into 0/1 = good/bad calculus score (cut-off point 0.7). The OHI-S was calculated by summing the debris and calculus scores (range 0-6). For the analysis, the OHI-S scores were dichotomized into 0 = good oral hygiene (OHI-S ≤ 1) and 1 = poor oral hygiene (OHI-S > 1). Occlusion was recorded according to Björk et al. [27], as modified by al-Emran et al. [28]. A sum score for malocclusions (SMO) was calculated based on a diagnosis of the absence (0)/presence (1) of the following phenomena: maxillary overjet, mandibular overjet, class II or class III molar occlusion, open bite, deep bite, lateral cross bite, midline shift, scissors bite, crowding, or spacing. Detailed information about the criteria used for the single malocclusion diagnoses are presented in a previous study [20].

### Reproducibility and internal consistency reliability

In Dar es Salaam and Arusha, duplicate clinical examinations were carried out on randomly selected sub-samples of 71 and 25 individuals, respectively, considered to be representative of the study subjects. In Dar es Salaam, the kappa statistics were 0.93 for the decayed, missed and filled teeth (DMFT) scores, 0.74 for the OHI-S scores, 0.78 for the midline shift scores, 0.79 for the deep bite scores, 0.82 for the mandibular overjet scores, 0.93 for the maxillary overjet scores, and 0.97 for the spacing scores. The kappa statistics were 1 for the scores for open bite, angle classification, cross bite, scissor bite, and crowding. The test-retest reliability for the eight Child-OIDP items ranged from 0.7 (emotional state) to 1.00 (eating, speaking, cleaning teeth, sleeping, smiling, and social contact). In Arusha, the kappa statistics were 0.78, 0.67, and 0.83 for the calculus, OHI-S, and DMFT scores, respectively. These figures indicate good and very good intra-examiner reliability [25]. The internal consistency reliability (standardized item α) of the Child-OIDP inventory was 0.85 in Arusha and 0.77 in Dar es Salaam, which agree with the values obtained previously in Tanzania [see [16,20]].

### Statistical analysis

Statistical Package for the Social Sciences (SPSS) version 15.0 was used for the data analysis. We adjusted for the design effect at both sites using STATA 10.0. The discriminative abilities of the generic and CS Child-OIDP scores were examined by comparing the distributions of both scores between groups with various levels on clinical indicators. Bivariate analyses of the Child-OIDP prevalence scores were conducted using cross-tabulations and χ^2 ^statistics. The overall generic and CS Child-OIDP scores were not normally distributed and the clinical groups were compared using the Mann-Whitney *U *test. To interpret the mean differences in scores across groups, the effect sizes were calculated as the mean differences between groups divided by the pooled standard deviations. The widely accepted thresholds of 0.2, 0.5 and 0.8 were used to define small, moderate, and large effect sizes [29]. Comparison of the generic and CS Child-OIDP attributed to dental caries, periodontal problems, and malocclusion were evaluated with Cochran's Q (for prevalence) and Friedman's test (for the overall scores) for related samples. Multiple-variable analyses were conducted using standard logistic regression with odds ratios (ORs) and 95% confidence intervals (CIs).

## Results

### Sample characteristics

As shown in Table 1, the percentage distribution of the participants' socio-demographic data and generic Child-OIDP scores varied systematically according to the study site. In Arusha, the study group of 1077 secondary school children (response rate, 80.9%) had a mean age of 14.98 years (SD 1.4), and included 46.6% boys. The mean OHI-S scores were 1.1 (SD 0.8), and the prevalence of poor oral hygiene (OHI-S > 1) was 44.8%. The mean DMFT was 1.2 (SD 1.8) and the prevalence of caries (DMFT > 0) was 43.5%. In Dar es Salaam, the study group of 1601 primary school students had a mean age of 13.0 years and comprised 39.5% boys. The mean DMFT score was 0.38 (SD 0.85), caries prevalence was 22.0%, the mean OHI-S score was 1.1 (SD 0.5), and the prevalence of OHI-S scores > 1 was 45.3%. The mean sum malocclusion score (SMO) was 1.1 (SD 1.0) and the prevalence of malocclusion was 63.8%. Midline shift (22.5%), spacing of at least 2 mm (21.9%), open bite (16.1%), and maxillary overjet were the most commonly diagnosed malocclusions, and mandibular overjet ≥ 2 mm (0.2%), cross bite (5.1%), and sagittal molar relationship class III (2.0%) were the least commonly diagnosed malocclusions [20].

**Table 1:** Percentage distributions (n) of participants by socio-demographic and clinical characteristics and study site

	Arusha % (n)	Dar es Salaam % (n)
*Sex*		
Male	46.6 (502)	39.5 (632)
Female	53.4 (575)	60.5 (969)**
*Age*		
Younger (12-13 yr)	12.3 (132)	69.6 (1115)
Older (≥ 14 yr)	87.7 (945)	30.4 (486)**
*Religious affiliation*		
Christian	84.7 (877)	44.4 (711)
Other	15.3 (148)	55.6 (890)**
*Residence*		
Urban	40.7 (438)	70.5 (1129)
Rural	59.3 (639)	29.5 (472)**
*Oral hygiene status*		
Good (OHI-S ≤ 1)	55.2 (594)	54.7 (876)
Poor (OHI-S > 1)	44.8 (483)	45.3 (725)
*Caries experience*		
DMFT = 0	56.5 (609)	78.0 (1249)
DMFT > 0	43.5 (468)	22.0 (352)
*Generic Child-OIDP*		
No impact (OIDP = 0)	49.3 (509)	71.4 (1143)
Impact (OIDP > 0)	50.7 (524)	28.6 (458)**

** *P *< 0.001

### Comparing the discriminative ability of the generic Child-OIDP across study sites

Statistically significant differences were observed in the prevalence and overall generic Child-OIDP mean scores between students with and without caries and with and without poor oral hygiene (Table 2). The effect sizes of the mean differences in the generic Child-OIDP scores between groups without and with dental caries were 0.3 (mean 1.3, SD 1.9 without caries; mean 2.0, SD 2.4 with caries) and 0.2 (mean 0.5, SD 1.1 without caries; mean 0.8, SD 1.4 with caries) in Arusha and Dar es Salaam, respectively. The corresponding effect sizes between the groups with and without a treatment need for periodontal problems were 0.2 (mean 1.3, SD 2.0 in children with a good OHI-S score; mean 1.9, SD 2.3 in children with a poor OHI-S score) and 0.1 (mean 0.5, SD 1.1 in children with a good OHI-S score; mean 0.7, SD 1.3 in children with a poor OHI-S score; not shown in Table 2). A multiple-variable logistic regression analysis was conducted with the generic Child-OIDP scores as the dependent variable and the DMFT and OHI-S scores as the independent variables, while adjusting for study site and potentially confounding socio-demographic factors. The interaction effects between the clinical indicators and the study sites were not statistically significant, suggesting that the discriminative capacity of this index with respect to dental caries and periodontal problems did not vary between the study sites. The site-specific OR estimates with DMFT > 0 were 1.6 (95% CI 1.3-1.9) in Arusha and 1.5 (95% CI 1.2-2.1) in Dar es Salaam. The corresponding ORs when OHI-S scores > 1 were 1.6 (95% CI 1.1-2.0) in Arusha and 1.2 (95% CI 1.0-1.5) in Dar es Salaam (not shown in Table 2).

**Table 2:** Discriminative capacity of the generic Child-OIDP for school children with and without normative treatment needs for dental caries or periodontal problems across the Arusha and Dare es Salaam study sites

	mean (SD) [effect size]	% (n)	Adjusted OR (95% CI)
***Dental caries***			
DMFT = 0	0.8 (1.5)	32.7 (601)	1
DMFT > 0	1.5 (2.1)** [0.4]	47.8 (381)**	1.5 (1.3-1.9) ^a^
***Periodontal***			
OHI-S < 1 (good)	0.8 (1.6)	33.9 (491)	1
OHI-S > 0 (poor)	1.2 (1.8)** [0.2]	41.4 (491)**	1.6 (1.2-1.6) ^a^

^a ^ORs for generic Child-OIDP adjusted for study site, age, sex, urban/rural residence, and religion

**P < 0.001, *P < 0.05

### Comparing the discriminative ability of the generic and CS Child-OIDP inventories

When the generic Child-OIDP was used, statistically significant differences in the overall mean scores were observed between the groups with and without decayed teeth, missing teeth and poor plaque scores. The corresponding effect sizes of the mean differences were 0.2, 0.2, and 0.2, respectively. As shown in Table 3, there were corresponding statistically significant differences between the groups in the prevalence of the generic Child-OIDP. The adjusted ORs for the association between decayed teeth (DT > 0) and the generic Child-OIDP score was 1.5. The corresponding figure for the association between a poor plaque score and the generic Child-OIDP score was 1.3. As shown in Table 4, there were significant differences in the overall scores between the groups with and without DMFT > 0, with and without DT > 0, and with and without missed teeth (MT > 0) when the CS Child-OIDP attributed to dental caries was used. The corresponding effect sizes were 0.8, 0.7, and 0.7, respectively. There were also significant differences in the overall mean scores between the groups that did and did not require normative treatment for malocclusion when the CS Child-OIDP attributed to malocclusion was used. The effect sizes ranged from 0.1 (open bite, midline shift, and the summed malocclusion score) to 0.5 (crowding). The adjusted ORs for the association between normative treatment of dental caries and the CS Child-OIDP attributed to dental caries were 5.4, 4.7, and 4.2 with respect to DMFT, DT, and MT, respectively. The adjusted ORs for the association between the normative treatment of malocclusion and the CS Child-OIDP attributed to malocclusion ranged from 2.5 (midline shift) to 8.8 (crowding).

**Table 3:** Generic Child-OIDP in children from Dar es Salaam with and without various types of normative treatment needs

	Mean OIDP (SD)	**Effect size**^**§**^	OIDP > 0% (n)	OIDP = 0% (n)	Adjusted OR (95% CI)
*Dental caries*					
DT = 0	0.5 (1.2)		27.0 (362)	73.0 (979)	1
DT > 0	0.8 (1.4)*	0.2	36.9 (96)**	63.1 (164)	1.5 (1.2-2.1) ^a^
MT = 0	0.6 (1.2)		28.2 (406)	71.8 (1036)	1
MT > 0	0.8 (1.5)*	0.2	32.7 (52)	67.3 (107)	1.2 (0.8-1.8) ^a^
*Periodontal*					
Plaque: good (PL score < 0.7)	0.5 (1.1)		24.8 (184)	75.2 (557)	1
Plaque: poor (PL score ≥ 0.7)	0.7 (1.3)**	0.2	31.9 (174)**	68.1 (586)	1.3 (1.1-1.7) ^a^
Calculus: good (calc. < 0.7)	0.6 (1.2)		28.1 (396)	71.9 (1012)	1
Calculus: poor (calc. score ≥ 0.7)	0.7 (1.4)	0.1	32.1 (62)	67.9 (131)	1.2 (0.8-1.6) ^a^
*Malocclusion*					
SMO = 0 (at least one malocclusion diagnosed)	0.6 (1.3)		27.4 (155)	72.6 (411)	1
SMO > 0 (more than one malocclusion diagnosed)	0.6 (1.3)	0.01	29.3 (303)	70.7 (732)	1.1 (0.8-1.1) ^a^
Open bite: absent	0.6 (1.2)		28.3 (381)	71.7 (963)	1
Open bite ≥ 2 mm	0.7 (1.3)	0.1	30.0 (77)	70.0 (180)	1.1 (0.8-1.5) ^a^
Maxill. overjet: absent	0.6 (1.2)		29.0 (341)	71.0 (833)	1
Maxill. overjet: ≥ 5 mm	0.5 (1.2)	0.1	22.7 (42)	77.3 (143)	0.7 (0.4-1.0) ^a^
Mand. overjet: absent	0.6 (1.2)		28.6 (419)	71.4 (1047)	1
Mand. overjet: > 0 mm	0.6 (1.3)	0.0	28.9 (39)	71.1 (96)	1.0 (0.7-1.5) ^a^
Midline shift: absent	0.6 (1.2)		28.1 (348)	71.9 (892)	1
Midline shift: ≥ 2 mm	0.7 (1.3)	0.1	30.5 (110)	69.5 (251)	1.1 (0.8-1.4) ^a^
Crowding: absent	0.6 (1.2)		28.4 (391)	71.6 (985)	1
Crowding: present	0.6 (1.2)	0.0	29.8 (67)	70.2 (158)	1.0 (0.7-1.4) ^a^

^a^Adjusted for study site and socio-demographic factors, such as age, sex, residence, and religion

**P < 0.001, *P < 0.05

^§^Effect size of mean differences

**Table 4:** CS Child-OIDP scores for dental caries, periodontal disease, and malocclusion in children from Dar es Salaam with and without various types of treatment needs

	Mean CS OIDP (SD)	**Effect size**^**§**^	OIDP > 0% (n)	OIDP = 0% (n)	Adjusted OR (95% CI)
*Dental caries*					
DMFT = 0	0.1 (0.5)		7.8 (97)	92.2 (1152)	1
DMFT > 0	0.7 (1.2)**	0.8	31.3 (110)**	68.8 (242)	5.4 (3.9-7.3) ^a^
DT = 0	0.1 (0.5)		9.2 (123)	90.8 (1218)	1
DT > 0	0.7 (1.3)**	0.7	32.3 (84)**	67.7 (176)	4.7 (3.4-6.5) ^a^
MT = 0	0.2 (0.6)		10.6 (153)	89.4 (1289)	1
MT > 0	0.7 (1.3)**	0.7	34.0 (54)**	66.0 (105)	4.2 (2.9-6.2) ^a^
*Periodontal*					
OHI-S < 1.0 (good)	0.3 (0.9)		14.1 (148)	85.9 (904)	1
OHI-S ≥ 1.0 (poor)	0.4 (0.9)		14.2 (78)	85.8 (471)	0.9 (0.7-1.3) ^a^
Plaque: good (PL < 0.7)	0.2 (0.8)		13.0 (96)	87.0 (645)	1
Plaque: poor (PL ≥ 0.7)	0.4 (1.0)*	0.1	15.1 (130)		1.2 (0.8-1.2) ^a^
Calculus: good (calc. < 0.7)	0.3 (0.8)		14.1 (198)	85.9 (1210)	1
Calculus: poor (calc. ≥ 0.7)	0.4 (1.0)		14.5 (28)	85.5 (165)	0.6 (0.1-1.5) ^a^
*Malocclusion*				84.9 (730)	
SMO = 0 (at least one malocclusion diagnosed)	0.01 (0.2)		0.4 (2)	99.6 (564)	1
SMO > 0 (more than one malocclusion diagnosed)	0.07 (0.5)*	0.1	3.6 (37)**	96.4 (998)	10.9 (2.6-45.8) ^a^
Open bite: absent	0.04 (0.3)		2.2 (29)	97.8 (1315)	1
Open bite: ≥ 2 mm	0.07 (0.4)	0.1	3.9 (10)	96.1 (247)	1.9 (1.0-4.0) ^a^
Maxill. overjet: absent	0.03 (0.3)		1.4 (17)	98.6 (1157)	1
Maxill. overjet: ≥ 5 mm	0.2 (0.6)**	0.3	7.0 (13)**	93.0 (172)	5.4 (2.5-11.6) ^a^
Mand. overjet: absent	0.04 (0.3)		2.0 (30)	98.0 (1436)	1
Mand. overjet: > 0 mm	0.2 (0.6)*	0.2	6.7 (9)*	93.3 (126)	3.2 (1.5-7.1) ^a^
Midline shift: absent	0.04 (0.3)		1.9 (23)	98.1 (1217)	1
Midline shift: ≥ 2 mm	0.09 (0.5)**	0.1	4.4 (16)*	95.6 (345)	2.5 (1.3-4.9) ^a^
Crowding: absent	0.02 (0.2)		1.2 (17)	98.8 (1359)	1
Crowding: present	0.2 (0.7)**	0.5	9.8 (22)**	90.2 (203)	8.8 (4.5-16.9) ^a^

^a^Adjusted OR for study site and socio-demographic factors, such as age, sex, residence, and religion

**P < 0.001, *P < 0.05

^§^Effect size of mean differences

Table 5 shows the sample distributions according to the generic Child-OIDP and CS Child-OIDP scores for dental caries, periodontal problems, and malocclusion. The overall scores and the prevalence scores for oral impact were significantly lower when the CS Child-OIDP was used than when the generic Child-OIDP was used.

**Table 5:** Dar es Salaam sample distribution by generic OIDP and CS OIDP scores for dental caries, periodontal disease, and malocclusion

Indicator	Generic OIDP	CS OIDP caries	CS OIDP periodontal disease	CS OIDP malocclusion
*Mean*	0.6	0.2	0.4	0.1**^b^
SD	1.2	0.8	0.9	0.4
Min. value	0	0	0	0
Max. value	8	8	6	3
Prevalence of impact (OIDP > 0)				
Number of cases	458	207	304	44
Percentage of cases	28.6	12.9	19.0	2.7**^a^

^a^Cochran's Q *P *< 0.001

^b^Friedman *P *< 0.001

## Discussion

The assessment of OHRQoL in children is a relatively recent initiative and CS measures are yet to be applied [30-32]. Because of the plethora of oral conditions that affect the quality of children's lives, the issue of describing the CS impact has remained a challenge [1]. This study assessed for the first time the discriminative ability of the generic Child-OIDP across various socio-cultural contexts in Tanzania, and compared the discriminative abilities of the generic and CS Child-OIDP inventories with respect to normative treatment needs.

About half the school children in Arusha reported experience with any oral impacts on daily performances. This rate is higher than those reported previously in similarly aged groups of Tanzanian school children, but lower than those observed in Uganda and other developing countries [33-35]. Not unexpectedly, the younger primary school children in Dar es Salaam had less caries experience and a lower prevalence of impacts as assessed by the generic Child-OIDP than their older counterparts in Arusha. Nevertheless, the performance of the generic Child-OIDP inventory in distinguishing between subjects with and without dental caries and periodontal problems did not vary across the study sites. Both the overall means and the generic prevalence scores revealed that oral problems had a greater impact on children suffering caries and periodontal problems than on their counterparts without these problems. This supports the construct validity of the Child-OIDP when used in Tanzanian school children. Although the generic Child-OIDP scores are less comparable to the specific normative treatment needs for dental caries and periodontal problems, the positive association observed might be explained by inferring that dental caries and periodontal problems contribute greatly to the burden of oral impacts on children's quality of life. In a previous study, toothache was recognized as the main cause of six of eight performance impacts of school children in Kinondoni district and four of eight impacts of school children in Temeke district, in Dar es Salaam [13]. A mouth ulcer and bleeding and swollen gums were among the causes most frequently listed by those school children [13]. Studies conducted elsewhere have shown similar results. Oral conditions related to dental caries, such as toothache and sensitive teeth, had the greatest reported impact on the quality of life in 11-12-year-old children from developing countries [13,36]. Despite differences in the prevalence of Child-OIDP and in the modes of administering the inventory across the study sites, neither the discriminative capacity of the generic instrument with respect to dental caries and periodontal problems nor its internal consistency (reliability) varied across the study sites. Previous studies that compared self- and interviewer-administered Child-OIDP inventories in the same study group found that the instrument showed acceptable psychometric properties irrespective of the mode of its administration [37,38].

As shown in Table 3,4 and 5, the prevalence of oral impact obtained with the generic Child-OIDP was higher than that obtained with the CS Child-OIDP. Both the generic and CS Child-OIDP rates were relatively low compared with those obtained in children using other OHRQoL instruments. This might be attributable to the fact that the ultimate impacts assessed by OIDP are rare in most study populations [30]. From the overall mean scores and the prevalence scores, both the generic and CS Child-OIDP inventories indicated that children with caries, periodontal problems, or malocclusion experienced a greater oral impact than those without these conditions. This corroborates previous studies that showed that children suffering from various dental diseases and clinical symptoms have a poorer OHRQoL [13,33]. Using the thresholds defined by Cohen [29], the effect sizes for the generic Child-OIDP were small when children with normative treatment needs for dental caries and periodontal problems were compared with those without such treatment needs, and were almost negligible when children with and without orthodontic treatment needs were compared. In contrast, the effect sizes related to the mean differences in the CS Child-OIDP scores were negligible when children with and without periodontal problems were compared, moderate when children with and without malocclusion were compared, and large when children with and without dental caries were compared. The present findings agree with those of previous studies [6,14], indicating that the two forms of the Child-OIDP are complementary rather than alternative sources of information. Nevertheless, the CS Child-OIDP was better suited than the generic Child-OIDP to identifying school children according to their normative treatment needs for malocclusion and dental caries. When assessing the strength of the association between the clinical indicators and the prevalence of oral impact, the ORs were larger when the CS Child-OIDP attributed to dental caries and malocclusion was used than when the generic Child-OIDP was used, even after adjustments were made for socio-demographic factors (Tables 3 and 4). This finding corroborates some previous studies but is inconsistent with others. A recent study of Thai school children revealed that the generic and CS Child-OIDP inventories distinguished equally well the groups with and without normative treatment needs for dental caries [14]. Comparing the generic and CS Child-OIDP assessments of malocclusion in Brazilian adolescents, Bernabé [6] found that both inventories were able to discriminate between subjects with and without treatment needs. However, the CS Child-OIDP showed the largest effect size and therefore appeared to be the form best able to differentiate between groups of adolescents. Other studies have compared the discriminative abilities of generic HRQoL and OHRQoL instruments with respect to early childhood caries and found that the latter oral-specific instruments discriminated the clinical groups more efficiently [8].

It should be noted that the two study groups considered were not age and sex matched, nor were they comparable with respect to their other socio-demographic characteristics (Table 1). The age and sex distributions of the school children with and without dental caries, periodontal problems, and malocclusions also differed, and might therefore have confounded the associations between the normative treatment needs or clinical indicators and the prevalence of oral impacts. Most of the confounding effects were probably accounted for when the site-specific multivariable analysis was adjusted for age, sex, and other socio-demographic factors. A comparison of the sample characteristics of the Dar es Salaam participants with the corresponding child population statistic on markers of gender and parental education suggested that this sample was representative of the populations of children aged 12-14 years in the two districts investigated. No similar analysis of the school children in Arusha was performed. Although both samples were randomized cluster samples, the possibility of selection bias cannot be overlooked. The structured self- and interviewer-administered questionnaires used in this study had certain limitations, with bias attributed to social desirability, acquiescence, and lack of recall frequently encountered, particularly in the younger age groups [39]. Attempts were made to minimize these biases by informing the participants at both sites that their responses were confidential and that no-one could link their names to their responses. The estimates pertaining to the school children in Dar es Salaam might have been underestimated because social desirability bias is more pronounced with interviews than with self-administered questionnaires. Because the Child-OIDP was used as an interviewer-administered measure in Dar es Salaam, whereas the inventory was self-administered in Arusha, the comparability of the data collected across sites could be questioned [12,31,32]. Nevertheless, previous studies of children from the general population and from specific disease groups have supported the comparability of the two modes of administration of the Child-OIDP inventory [6,14].

## Conclusion

The generic Child-OIDP discriminated equally well between children with and without dental caries and periodontal problems across socio-culturally different study sites in Tanzania. Compared with its generic form, the CS Child-OIDP discriminated more effectively between children with and without dental caries or malocclusion. Thus, the CS Child-OIDP seemed to be better suited to support the clinical indicators of dental caries and malocclusion when the oral health needs of school children are estimated.

## Competing interests

The authors declare that they have no competing interests.

## Authors' contributions

HSM: principal investigator, designed the study, collected the data (Arusha study site), performed the statistical analyses, and wrote the manuscript. MT: investigated, designed, and collected the data at the Dar es Salaam site. JRM: participated in the design of the study and provided valuable guidance in the data collection at both sites, and has been actively involved in writing the manuscript. PD: supervised, designed, and provided guidance for the study at the Dar es Salaam site. ANÅ: main supervisor, designed the study, and guided the statistical analyses. All authors have read and approved the final manuscript.

## Pre-publication history

The pre-publication history for this paper can be accessed here:

http://www.biomedcentral.com/1471-2431/11/45/prepub
